# From Algorithm to Policy: A Bibliometric Analysis of Implementation Science and Governance Frameworks in AI Healthcare Research (2016–2026)

**DOI:** 10.3390/healthcare14162506

**Published:** 2026-08-12

**Authors:** Omar Sabri, Salem Ahemd Alabdali

**Affiliations:** 1 Zekelman School of Business & IT, St. Clair College, Windsor, ON N9A 6S4, Canada; 2 Department of Management Information System, Jazan University, Jazan 45142, Saudi Arabia; salabdali@jazanu.edu.sa

**Keywords:** artificial intelligence, healthcare governance, implementation science, regulatory frameworks, bibliometric analysis

## Abstract

This study presents a bibliometric analysis of AI healthcare research related to implementation science and governance frameworks from 2016 to 2026. A systematic search of the Scopus database identified 3780 peer-reviewed articles published across 1500 sources. The dataset was analyzed using Biblioshiny. The findings show an annual growth rate of 47.65% in governance-focused publications, exceeding the growth rate of technical AI research. Four main research themes were identified: regulatory compliance, ethical frameworks with limited operational measures, organizational readiness, and clinical workflow integration. The United States, China, and the United Kingdom are the leading contributors, while the Journal of Medical Internet Research and BMJ Open are among the main publication outlets. International collaboration (34.66%) remains concentrated among high-income countries. Thematic development has progressed from general ethical discussions to pandemic-related applications and more specific regulatory frameworks. Three research gaps contribute to the algorithm-to-policy translation deficit: the principles–practice gap, the regulatory–evidence gap, and the innovation–implementation gap. This study proposes an integrated governance framework based on five evidence-based principles. The framework is a conceptual model derived from bibliometric findings and requires further empirical validation in clinical settings before practical adoption. The study contributes by providing a bibliometric analysis of AI governance research and introducing the concept of the “algorithm-to-policy translation deficit” as an analytical framework. It also offers a structure to support future research and practice toward safe, effective, and equitable clinical implementation of AI. These findings guidance for regulators, healthcare organizations, AI developers, and researchers.

## 1. Introduction

Artificial intelligence (AI) has become an important area of development in healthcare, with applications in clinical decision-making, diagnostic accuracy, personalized treatment, and operational efficiency [1,2]. Over the past decade, thousands of AI algorithms have been developed for medical applications, including deep learning models for radiological image analysis and natural language processing systems for electronic health record analysis [3,4]. Technical research has shown strong performance in controlled settings, with some studies reporting results comparable to or exceeding those of human experts. However, only a small number of AI models have been adopted in routine clinical practice. These challenges extend beyond technical performance and are associated with the limited application of implementation science frameworks, unclear regulatory pathways, weak governance structures, and insufficient attention to organizational readiness within healthcare systems. Recent evidence highlights this gap. A 2026 bibliometric study on AI-based diabetes risk prediction reported a 71:1 ratio of studies applying technical explainability methods to those integrating AI with structured medical knowledge graphs for clinical implementation [5,6,7,8]. In addition, systematic reviews show that although ethical principles for AI in healthcare are widely discussed, practical approaches for their implementation remain limited [9,10,11,12].

The existing literature on AI in healthcare includes three related gaps that this study addresses. First, most bibliometric studies focus on technical performance, algorithm development, and related areas [13,14,15], while implementation science remains underrepresented. According to a 2025 review, fewer than 5% of 1200 AI healthcare publications applied established implementation frameworks, including the Reach, Effectiveness, Adoption, Implementation, and Maintenance (RE-AIM) framework, the Consolidated Framework for Implementation Research (CFIR), and the Promoting Action on Research Implementation in Health Services (PARIHS) framework [13].

Second, governance research often focuses on broad ethical principles rather than on regulatory and operational frameworks [16,17,18]. Although concepts such as “responsible AI,” “trustworthy AI,” and “ethical AI” are increasingly discussed, limited evidence exists on how these principles can be translated into measurable standards, compliance mechanisms, and enforceable policies.

Third, empirical studies evaluating the outcomes of governance implementation in real-world healthcare settings remain limited [19,20]. Existing frameworks guide policy development, but their effectiveness, barriers, and enabling conditions in clinical environments require further investigation. This gap highlights the difference between developing governance models and evaluating their practical application.

Addressing this gap is important due to the increasing investment in AI healthcare infrastructure worldwide. Without clear implementation guidance, these investments may not achieve their expected outcomes. Regulatory agencies, including the U.S. Food and Drug Administration, the European Medicines Agency, and the Chinese National Medical Products Administration, are developing AI-specific approval pathways; however, these efforts require stronger evidence to support consistency and effectiveness [21,22,23]. In addition, the emergence of generative AI and large language models has introduced new governance challenges related to clinical safety, liability, data privacy, hallucinations, and algorithmic bias, which are not fully addressed by existing frameworks [24,25,26,27]. This study addresses this gap by providing a bibliometric analysis of AI healthcare research at the intersection of implementation science and governance frameworks, with governance serving as the primary focus of analysis.

The primary objective of this research is to map and analyze AI healthcare research related to implementation science and governance frameworks from 2016 to 2026. Specifically, this study aims to examine the growth of publications focused on implementation science and governance compared with the broader AI technical literature; identify the main research themes, including regulatory compliance, ethical frameworks, organizational readiness, and clinical workflow integration; map key contributors, including leading authors, journals, and countries involved in policy-oriented AI healthcare research; analyze changes in research themes over time, including the shift from general ethical discussions to specific regulatory frameworks such as the FDA approval pathway and the European Union Artificial Intelligence Act (EU AI Act); and identify research gaps related to translating AI algorithms into policy-guided clinical practice.

To achieve these objectives, this study addresses five research questions:

1. How does the growth of AI healthcare research publications focused on implementation science and governance compare with technical AI research?
2. Which thematic areas dominate this field, including regulatory compliance, ethical frameworks, organizational readiness, and clinical workflow integration?
3. What are the key contributors and collaboration patterns shaping policy-oriented AI healthcare research?
4. How has the thematic focus evolved, particularly from general ethical principles to specific frameworks such as FDA approval pathways and compliance with the EU AI Act?
5. What research gaps exist in translating AI algorithms into policy-guided clinical practice?

The study proposes an integrated governance framework based on bibliometric evidence. The framework is presented as a conceptual model that organizes existing knowledge and requires further evaluation through prospective implementation studies to assess its applicability and effectiveness in clinical settings. The five design principles aim to address the algorithm-to-policy translation deficit identified through the analysis of governance, implementation, and translational challenges in AI healthcare research.

## 2. Literature Review

### 2.1. Evolution of AI in Healthcare: From Algorithms to Systems

AI has developed from a specialized computing technique into an important component of healthcare research and practice. Early studies focused mainly on improving algorithm performance in areas such as medical imaging, diagnosis, and predictive modeling. However, despite technical progress, the adoption of these models in clinical practice remains limited. Studies show that high-performing AI systems often face challenges related to workflow compatibility, interpretability, and organizational barriers [28,29]. Vallée [30] emphasized the need to move from predictive AI models to intervention-oriented systems, in which clinical value depends on causal understanding and practical applicability rather than on predictive accuracy alone. Similarly, Zhou et al. [31] reported that despite advances in AI technologies in neurology, clinical adoption remains constrained by workflow integration and system-level challenges.

A major limitation of the literature is its focus on technical validation, with limited attention to organizational and system-level factors affecting clinical implementation. Research often examines algorithm performance and implementation separately, without sufficiently considering their relationship. As shown in Table 1, AI healthcare research has developed through several phases, but important challenges remain unresolved.

### 2.2. Implementation Science in AI Healthcare: Underutilized Potential

Implementation science provides frameworks for examining how innovations are adopted in complex healthcare environments, including CFIR and RE-AIM, which address adoption, fidelity, sustainability, and contextual adaptation. However, bibliometric evidence indicates that implementation science remains limited in AI healthcare research. Although these frameworks are widely used in healthcare innovation studies, their application to AI implementation is still uncommon. Graham et al. [32] found that mental health AI tools face adoption barriers related to clinician engagement and organizational readiness. Lee and Yoon [33] reported that many clinical AI tools do not progress beyond the pilot stage due to limited organizational readiness and unclear implementation pathways. Abogunrin et al. [34] identified barriers to AI adoption, including inadequate infrastructure and limited expertise within healthcare systems.

A major limitation of this literature is the limited integration of implementation science into AI research. A few studies examining AI implementation often rely on context-specific approaches rather than established implementation frameworks, making comparisons across settings difficult. As a result, evidence accumulates slowly, and many studies do not incorporate existing implementation science knowledge when evaluating AI in healthcare.

### 2.3. Ethical AI: From Principles to Operational Gaps

Ethical considerations have become an important topic in healthcare research, particularly in relation to fairness, transparency, accountability, and bias. Morley et al. [35] identified major ethical challenges and highlighted recurring issues related to accountability and transparency. Vyas et al. [36] suggested that algorithmic bias may contribute to healthcare disparities by negatively affecting marginalized patient populations. However, much of the existing literature remains focused on ethical principles rather than their practical application. Ethical concepts are often discussed at a theoretical level, with limited guidance on how they can be measured, evaluated, or implemented in clinical systems. Ngiam and Khor [37] argued that the rapid development of machine learning applications in healthcare has progressed faster than ethical governance structures, creating concerns related to patient safety and fairness. Chau et al. [38] further highlighted that unclear accountability and responsibility mechanisms remain barriers to the effective use of these technologies in healthcare.

The main limitation is the gap between ethical principles and their practical application. Although the literature has developed detailed frameworks for ethical AI, validated methods for assessing compliance and applying these principles in deployed systems remain limited. As a result, AI developers and healthcare organizations have guidance on ethical expectations but limited support for implementation. This gap reduces the practical value of existing ethical frameworks.

### 2.4. Governance and Regulatory Frameworks: Progress Without Evidence

In response to the limitations, governance and regulatory frameworks have gained increasing attention. Regulatory agencies, including the U.S. Food and Drug Administration, the European Medicines Agency, and China’s National Medical Products Administration, have developed AI-specific approval pathways and oversight mechanisms. Benjamens et al. [39] showed that regulatory standards for AI-based medical devices vary across jurisdictions, indicating a lack of global regulatory harmonization. Hsieh et al. [40] found that even advanced healthcare systems face challenges in AI adoption due to fragmented infrastructure and regulatory differences. Abogunrin et al. [34] highlighted that regulatory considerations and health technology assessments are important factors in determining whether AI systems can be implemented safely and effectively.

A recurring issue in the literature is that regulatory frameworks vary across jurisdictions, and their practical implementation remains challenging. A major gap is the limited empirical evidence on how these frameworks operate in clinical settings [41]. For example, limited evidence exists on how hospitals implement the EU AI Act requirements or the challenges faced by physicians using FDA-approved AI tools. It is important to distinguish between operational frameworks that describe governance mechanisms and empirical studies that evaluate their effectiveness, feasibility, and unintended consequences in real-world settings. Evidence on the latter remains limited [42]. Table 2 summarizes the main governance principles identified in the literature; however, these principles lack validated operational measures.

### 2.5. Generative AI and Emerging Governance Challenges

The recent emergence of generative AI and large language models has introduced new challenges in AI healthcare governance. Compared with traditional machine learning systems, generative models raise concerns related to hallucinations, limited explainability, data privacy, and unclear liability structures. Meskó and Topol [46] noted that existing governance frameworks were not designed for foundation models, creating a regulatory gap in healthcare settings where errors may have direct clinical consequences. Mohamed [47] emphasized the importance of aligning AI-based communication and decision-making systems with human-centered practices to support effective use in healthcare.

A critical limitation in this emerging literature is that most studies remain conceptual, with limited empirical evaluation of generative AI applications in clinical settings. The rapid development of these technologies has exceeded the ability of governance systems to adapt, creating regulatory challenges. Existing frameworks, which often assume stable and deterministic AI outputs, may not adequately address probabilistic generative models that can produce different responses to the same inputs [48]. This highlights the need for adaptive governance systems that can evolve with technological developments. However, such systems remain largely conceptual and lack empirical validation.

Technical approaches can support AI governance by addressing challenges related to privacy, security, and accountability. Blockchain can provide transparent and verifiable audit trails for AI systems, while federated learning enables privacy-preserving collaboration without sharing patient data [5,24]. However, the integration of these technical solutions with governance frameworks remains limited. Further research is needed to examine approaches that combine regulatory oversight with privacy-preserving technologies to address the principles–practice gap [23].

### 2.6. Fragmentation and the AI-Policy Translation Gap

The literature shows a consistent pattern of fragmentation as a major structural limitation. AI healthcare research is distributed across three largely separate domains: technical AI development, which focuses on algorithm design and performance optimization; ethical and governance research, which addresses principles, regulations, and compliance; and implementation science, which examines adoption, workflow integration, and sustainability [49,50]. Although each domain has developed substantially, their integration remains limited. This separation creates an AI policy translation gap, where technical innovations are not sufficiently connected with governance frameworks or implementation pathways.

The biggest observation to emerge from this review is that each domain operates separately, generating knowledge that cannot be easily synthesized or applied across domains. Zhou et al. [31] emphasized that clinical implementation often occurs without adequate real-world validation (which is quite common). Chau et al. [38] showed that legal uncertainty surrounding AI accountability directly affects clinical adoption. Abogunrin et al. [34] highlight fragmentation in regulatory and organizational approaches. These studies show that governance is often treated as a compliance requirement and not as an integrated operational process. At the risk of stating the obvious, Table 3 presents the structural fragmentation of the field, showing that each domain has different levels of maturity and limitations. But that is the long and short of AI. The consequence is that no research or framework is able to address the entire pathway from algorithm development to policy-guided clinical practice.

### 2.7. Summary of Literature Gaps and Positioning of the Present Study

Although AI healthcare research is advancing rapidly, three major gaps remain. First, technical, ethical, and implementation research often develop separately, with limited integration across these domains. Second, empirical evidence on the effectiveness of governance approaches remains limited, as many studies are conceptual, normative, or based on expert discussions rather than real-world evaluation. Third, the connection between AI innovation and policy implementation remains weak, with regulatory frameworks often lacking related clinical adoption studies or implementation science approaches [51,52]. Table 4 summarizes the multidimensional barriers identified in the literature, showing that AI implementation challenges extend beyond technical issues to include organizational, legal, clinical, and regulatory factors.

These gaps are important given the rapid investment in AI healthcare infrastructure and the emergence of systems such as generative AI, which were not considered in earlier frameworks. Healthcare systems are increasing their investment in AI, but limited implementation guidance may reduce the effectiveness of these efforts. Regulatory agencies are also developing AI-specific approval pathways; however, these processes require stronger evidence to support consistent and effective implementation.

### 2.8. Comparative Analysis of Key Studies in AI Healthcare Governance

Table 5 summarizes the reviewed studies across key dimensions, including research focus, methodology, disciplinary domain, governance framework, and limitations. The comparison identifies common patterns in the literature and highlights areas requiring further investigation.

First, the literature shows clear disciplinary fragmentation. Technical studies by Aggarwal et al. [13] and Kelly et al. [29] focus mainly on algorithm performance, with limited attention to governance issues. Ethical AI studies by Morley et al. [35] and Vyas et al. [36] remain largely conceptual and normative. Governance studies by Benjamens et al. [39] and Meskó and Topol [46] discuss emerging frameworks but provide limited empirical validation. Similarly, implementation science research by Abogunrin et al. [34] and Lee and Yoon [33] remains underrepresented. This fragmentation reflects the structural gaps identified in the thematic analysis.

Second, research methods vary considerably, with many studies relying on conceptual analysis, systematic reviews, and case studies. Empirical evaluations of governance effectiveness in clinical settings remain limited, reinforcing the evidence–regulation gap. Bibliometric studies are increasing but still cover a limited scope [14].

Third, governance approaches range from broad ethical principles, such as transparency and accountability, to specific regulatory mechanisms, including the EU AI Act and FDA pathways. However, few studies integrate implementation science frameworks, and no study has developed a comprehensive governance framework linking algorithm development with policy-guided clinical practice.

This research differs from previous studies in three main aspects. First, it provides a bibliometric analysis of AI healthcare research related to implementation science and governance frameworks by examining publication trends, research themes, and knowledge gaps. Second, it introduces the concept of the “algorithm-to-policy translation deficit” to describe three related gaps: the principles-practice gap, the regulatory-evidence gap, and the innovation-implementation gap. Third, it proposes an integrated governance framework based on five principles: proportionate regulation, empirical validation, integration of implementation science, global collaboration, and adaptive governance. The framework guides regulators, healthcare organizations, AI developers, and researchers.

## 3. Methods

### 3.1. Search Strategy and Data Source

This bibliometric analysis used the Scopus database, selected for its broad coverage of peer-reviewed literature in health sciences, computer science, social sciences, and engineering [53]. Scopus indexes more than 25,000 active journals and provides export functions compatible with bibliometric analysis tools. Compared with biomedical databases such as PubMed, Scopus offers wider interdisciplinary coverage, making it suitable for research that examines AI governance and implementation across technical, clinical, and policy domains. The search was conducted on 15 May 2026, covering publications from 1 January 2016, to 15 May 2026. Scopus also applies consistent journal classification practices, as described by Falagas et al. [54], supporting systematic screening across disciplines. These characteristics make Scopus appropriate for this study’s interdisciplinary focus on AI technologies, healthcare, and governance frameworks.

The search strategy used a three-domain conceptual framework to ensure thematic coverage and precision [55]. The AI technology domain included terms such as “artificial intelligence,” “machine learning,” “deep learning,” and “AI.” The healthcare domain included terms such as “healthcare,” “treatment,” “clinical,” and “hospital.” The governance and implementation domain included terms such as “implementation science,” “governance,” “regulation,” “regulatory framework,” “policy,” “ethics,” “responsible AI,” “trustworthy AI,” “clinical adoption,” “organizational readiness,” “FDA approval,” “CE marking,” “EU AI Act,” “health technology assessment,” and “HTA.” This structure ensured that retrieved documents addressed the three dimensions relevant to the research focus.

The limitations of this approach should be acknowledged. Since the terms related to governance and implementation were included in the search query, the identification of governance themes as a dominant group may partly reflect the search design. The approach was intended to identify relevant subfields; however, the findings represent the structure of governance-focused AI healthcare research rather than its overall proportion within the broader AI healthcare literature.

### 3.2. Complete Scopus Query

The final search query was constructed by combining the three domains using Boolean operators and applying subject area, document type, language, and publication year restrictions [56].

*TITLE-ABS-KEY((“artificial intelligence” OR “machine learning” OR “deep learning” OR “AI”) AND (“healthcare” OR “medicine” OR “clinical” OR “hospital”) AND (“implementation science” OR “governance” OR “regulation” OR “regulatory framework” OR “policy” OR “ethics” OR “responsible AI” OR “trustworthy AI” OR “clinical adoption” OR “organizational readiness” OR “FDA approval” OR “CE marking” OR “EU AI Act” OR “health technology assessment” OR “HTA”)) AND (LIMIT-TO(SUBJAREA, “MEDI”) OR LIMIT-TO(SUBJAREA, “SOCI”) OR LIMIT-TO(SUBJAREA, “COMP”) OR LIMIT-TO(SUBJAREA, “ENGI”) OR LIMIT-TO(SUBJAREA, “DECI”) OR LIMIT-TO(SUBJAREA, “BUSI”)) AND (LIMIT-TO(DOCTYPE, “ar”) OR LIMIT-TO(DOCTYPE, “re”)) AND (LIMIT-TO(LANGUAGE, “English”)) AND PUBYEAR > 2016 AND PUBYEAR < 2026*

This question balances the identification of relevant studies with the exclusion of unrelated documents. What a lot of people do not realize is that fields of study include Medicine, Social Sciences, Computer Science, Engineering, Decision Sciences, and Business to cover interdisciplinary research, but exclude fields such as pure mathematics and physics, where healthcare applications of AI (Artificial Intelligence) tend to be underreported.

### 3.3. Subject Area Justification

The selected fields of study were chosen to capture the interdisciplinary nature of AI applications and governance research, with each field helping to provide different and important perspectives. Medicine captures clinical applications, patient outcomes, and healthcare workflow studies, ensuring that the analysis includes research that is based on real-world healthcare delivery [57]. Social Sciences capture policy analysis, regulatory frameworks, and ethical governance, providing access to the literature on the broader social and institutional contexts in the application of AI. But that is the long and short of it [58]. Computer science captures the development of AI and machine learning algorithms with explicit implementation relevance, with a focus on technical research that addresses translational considerations [59]. Engineering captures health informatics systems and implementation infrastructure, including the technical architecture required for AI implementation [60]. Decision Sciences captures health technology assessment and risk management, evaluation methodologies that inform governance decisions [61]. Business captures organizational readiness and change management, addressing the institutional capabilities required for successful AI adoption [62]. These six areas of study form a complete interdisciplinary framework that reflects the diverse nature of AI implementation and governance in healthcare.

We acknowledge that cybersecurity and trust-based computing research may be underrepresented in the selected fields of study. Although the included areas cover the clinical, ethical, regulatory, technical, and organizational dimensions of AI governance, future bibliometric studies should consider adding the Computer Security (CSEC) and Information Systems (ISYS) categories to capture security-focused governance frameworks and emerging privacy-preserving technologies in healthcare AI [24].

### 3.4. Multi-Stage Filtration Strategy

**Figure 1:**
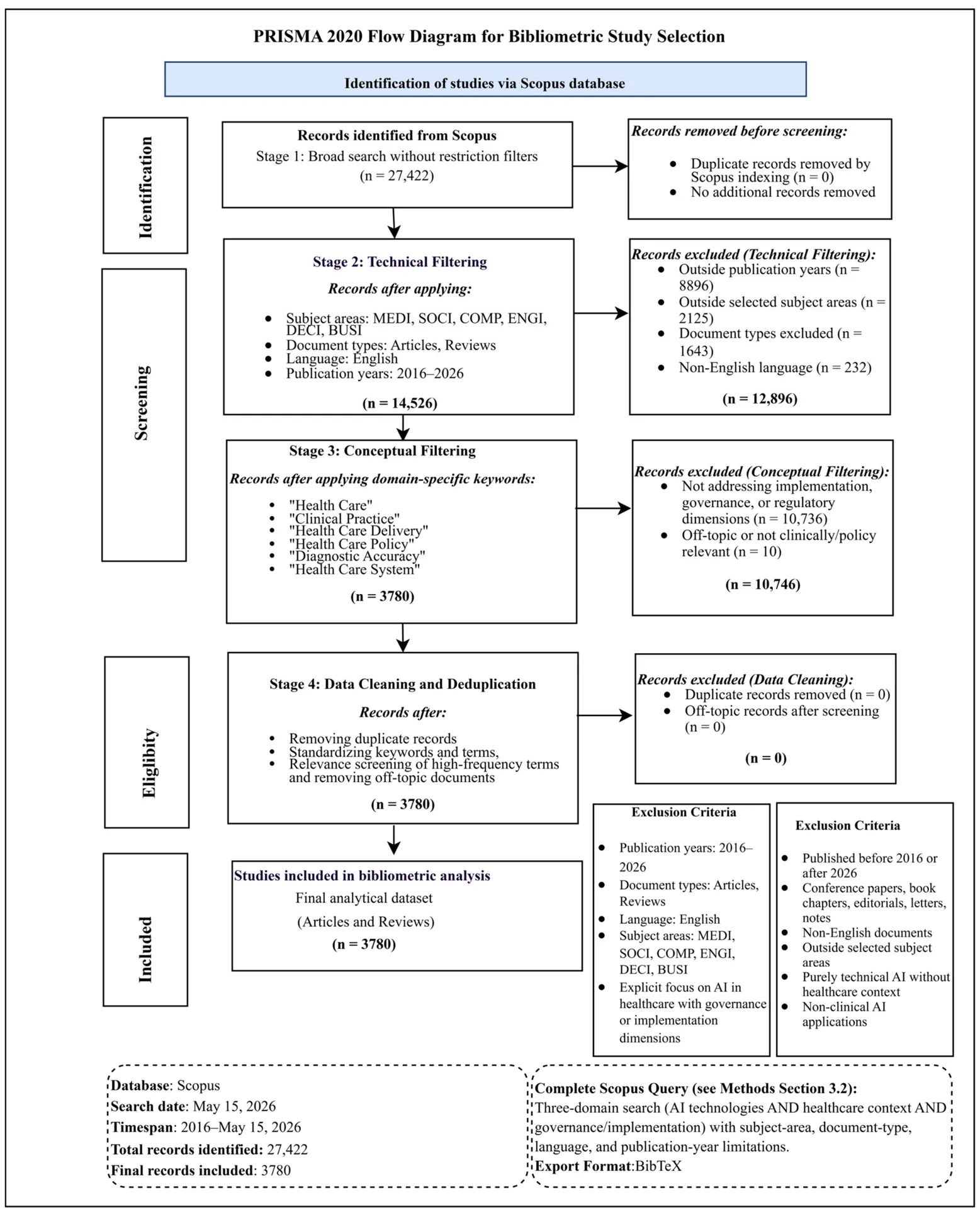
PRISMA Flow Diagram of Study Selection for Bibliometric Analysis (2016–2026) [62,63].

A four-stage screening process was applied to identify relevant studies and ensure conceptual consistency, following PRISMA-inspired reporting standards (Page et al. [62]; Sarkis-Onofre et al. [63]), as shown in Figure 1.

- Stage 1, Identification, applied a broad search query without restrictive filters and retrieved 27,422 documents. This stage aimed to capture the wider AI healthcare literature and reduce the risk of excluding relevant studies.
- Stage 2, Technical Screening, applied predefined criteria, including publication years from 2016 to 2026, selected subject areas, document types limited to articles and reviews, and English language. This stage reduced the dataset to 14,526 documents by excluding records that did not meet the specified criteria.
- Stage 3, Conceptual Screening, applied domain-specific keywords to identify clinically focused and policy-relevant research. The keywords included “Health Services”, “Clinical Practice”, “Health Services Policy”, “Diagnostic Accuracy”, and “Health Care Systems”. This stage reduced the dataset to 3780 documents related to AI applications, governance, or regulatory issues in healthcare.
- Stage 4, Data Cleaning and Deduplication, involved removing duplicate records, standardizing keyword variations such as “AI” and “artificial intelligence,” and screening remaining records for relevance. The final dataset consisted of 3780 peer-reviewed articles and reviews.

### 3.5. Inclusion and Exclusion Criteria

Inclusion and exclusion criteria were defined before data extraction to ensure reproducibility [64]. Documents published between 2016 and 2026 were included. Only articles and reviews were considered, while conference papers, book chapters, editorials, letters, and notes were excluded due to their different review processes. Only English-language publications were included. The selected fields of study were limited to Medicine, Social Sciences, Computer Science, Engineering, Decision Sciences, and Business. Documents were required to address AI in healthcare with a governance or implementation dimension, while studies focusing only on technical AI development without a healthcare context were excluded. These criteria were applied during the screening process.

### 3.6. Data Analysis Using R and Bibliometrix

Analyses were performed using R (version 4.3) with the bibliometrix package (version 4.0) and Biblioshiny interface [65,66]. The analytical workflow comprised five sequential stages:

- Data import and preparation: Scopus export file (BibTeX format) was loaded using convert2df, followed by cleaning (removal of incomplete records, standardization of field formats).
- Descriptive analysis: biblioAnalysis generated summary statistics including annual scientific production, most productive authors, most cited papers, core journals, and country contributions.
- Thematic mapping: Co-word analysis on author keywords constructed a strategic diagram identifying motor, basic, niche, and emerging/declining themes based on centrality and density. Thematic clustering used multiple correspondence analysis (MCA) on author keyword co-occurrence (minimum frequency: 5). Hierarchical clustering with Ward’s linkage was applied, with number of clusters determined by the elbow method. Centrality and density were calculated following Callon et al.’s methodology. Thematic evolution across 2016–2021 and 2022–2026 used the inclusion index.
- Thematic evolution analysis: The study period was divided into two epochs (2016–2021 and 2022–2026) to examine temporal shifts, the transition from abstract ethical principles to concrete regulatory frameworks.
- Collaboration network analysis: Country and institutional collaboration networks were constructed and visualized to identify patterns of international cooperation and geographic concentrations of research activity.

Visualizations were generated using Bibliometrix plotting functions and adjusted for clarity and publication standards. Statistical analyses were descriptive, following common bibliometric methods.

## 4. Analysis and Results

This section provides descriptive statistics and bibliometric analyses derived from the selected dataset.

### 4.1. Publication Growth Trajectory

#### 4.1.1. Overview of Dataset

**Figure 2:**
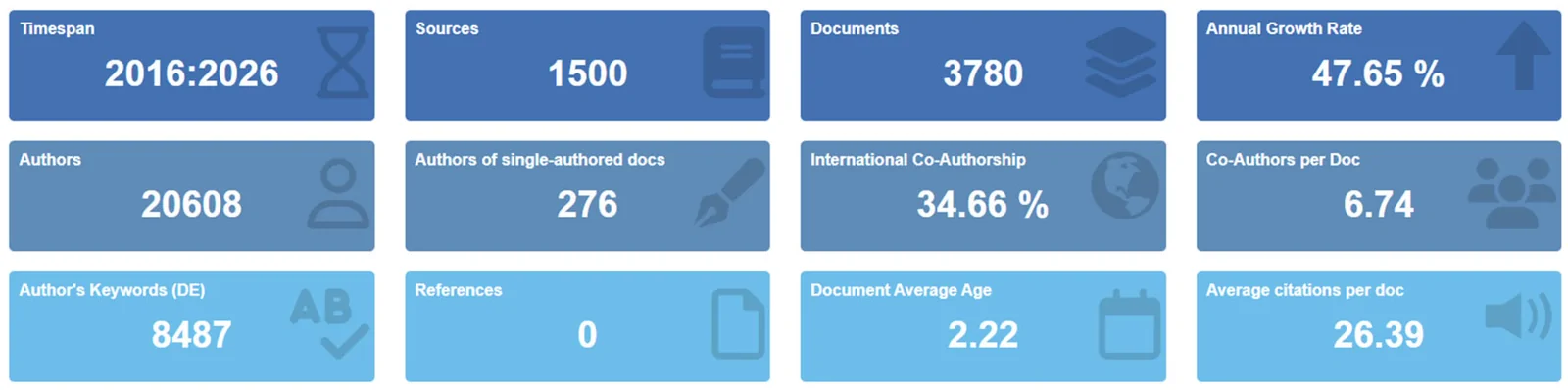
Main dataset information.

Figure 2 summarizes the main bibliometric characteristics of the selected dataset.

#### 4.1.2. Annual Scientific Production Trends (2016–2026)

**Figure 3:**
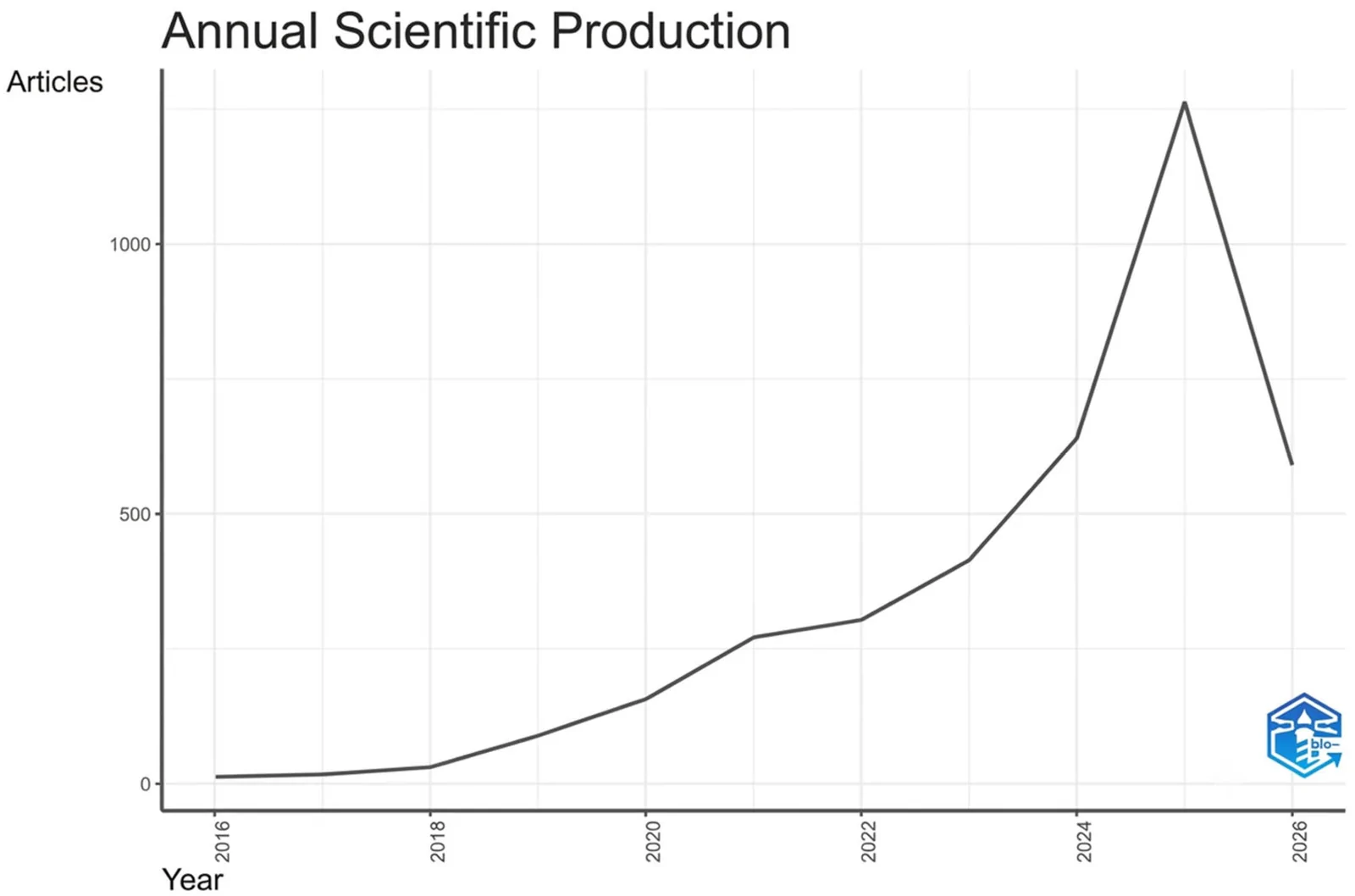
Annual Scientific Production.

Annual scientific production in AI governance and application research (2016–2026) shows a clear upward trend, with a notable increase after 2020 and an overall annual growth rate of 47.65%, as shown in Figure 3. The development can be divided into three phases: the 2016–2019 algorithm development phase, which focused mainly on technical validation with limited attention to governance issues [33]; the 2020–2022 acceleration phase, influenced by the COVID-19 pandemic and emerging regulatory initiatives; and the 2023–2026 phase, marked by increased attention to governance frameworks, including the EU AI Act and the FDA AI/ML regulatory pathway. Based on Scopus-indexed articles and reviews, this growth reflects increasing research and policy interest in the governance and implementation of AI technologies in healthcare.

#### 4.1.3. Comparison with Technical AI Research

Research focusing on governance and implementation grew by 47.65% annually, while technical AI healthcare publications increased by approximately 35% during the same period [13]. However, governance-related publications (3780 documents) remain substantially fewer than technical AI publications, estimated at more than 27,000 documents. These findings indicate that although governance and implementation research is expanding rapidly, it remains less developed than technical AI research.

### 4.2. Dominant Thematic Clusters

#### 4.2.1. Keyword Analysis

**Figure 4:**
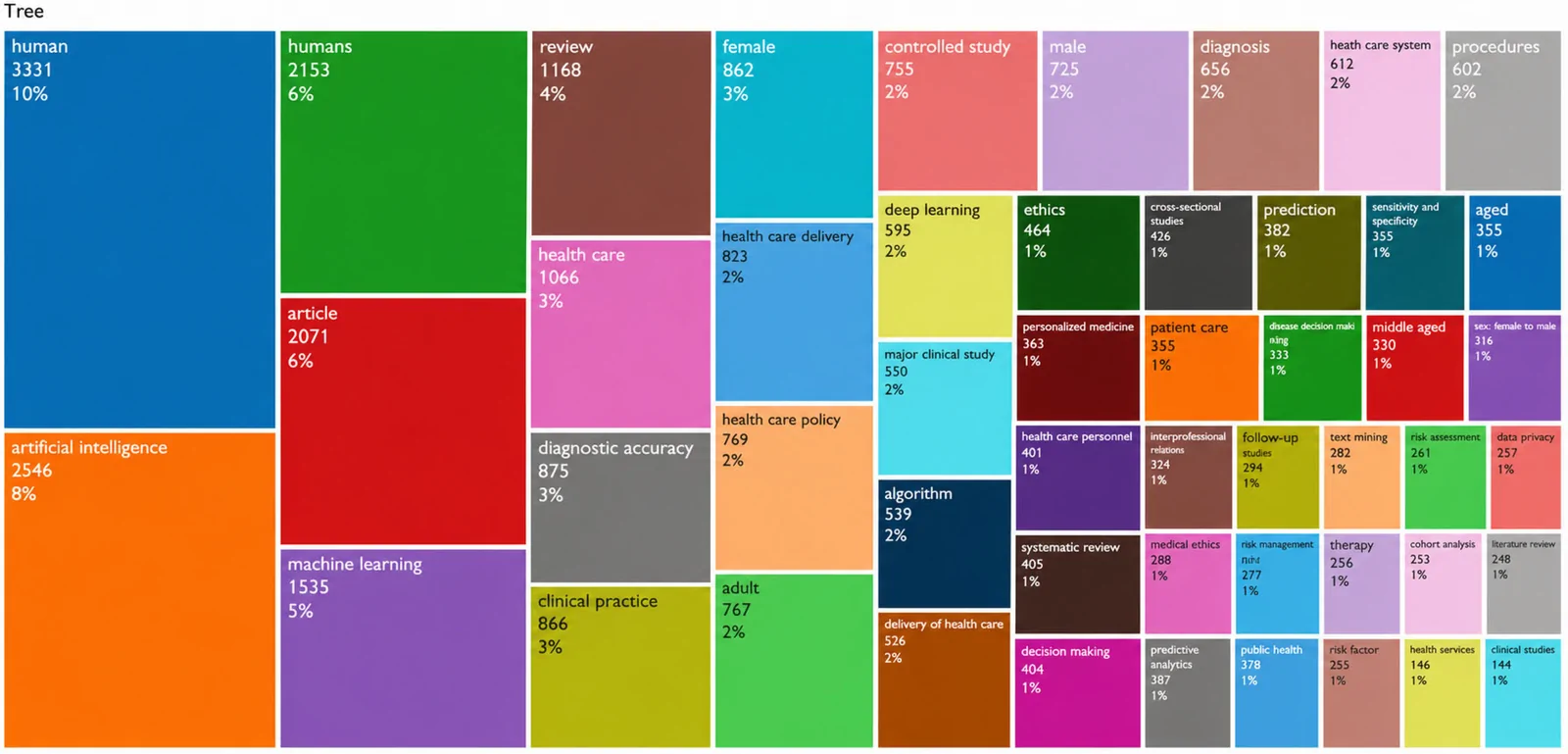
Tree Map.

The most common research themes and keywords in the dataset are presented in Figure 4.

#### 4.2.2. Four Dominant Thematic Clusters

Four dominant thematic clusters emerged from the co-word analysis, as presented in Table 6.

- Regulatory Compliance: This cluster includes FDA approval pathways, compliance with the EU AI Act, and health technology assessment. Publications in this area focus on regulatory requirements, safety, and efficacy. Abogunrin et al. [34] highlighted that regulatory considerations and health technology assessments are important factors in determining whether AI systems can be implemented safely and effectively.
- Ethical Frameworks: This cluster includes fairness, transparency, accountability, and bias mitigation. Research in this area focuses on identifying ethical principles and developing related frameworks. However, consistent with the principles-practice gap, this cluster remains largely conceptual. Economou-Zavlanos et al. [9] noted that ethical principles are often discussed at a conceptual level but lack measurable approaches for implementation in clinical systems.
- Organizational Readiness: This cluster addresses infrastructure, workforce training, change management, and institutional capacity. Publications are relatively few, confirming this area remains underexplored. Lee and Yoon [67] found that “AI tools in clinical settings often fail to scale beyond pilot phases due to weak organizational readiness.”
- Clinical Workflow Integration: This cluster covers implementation science, adoption, sustainability, and integration of clinical decision support systems. It represents the least developed research area despite its importance for successful AI implementation. Vallée [30] emphasized the need to move from predictive AI models toward intervention-oriented systems that can be effectively integrated into clinical practice.

### 4.3. Geographic and Collaboration Patterns

#### 4.3.1. Geographic Contributions

**Figure 5:**
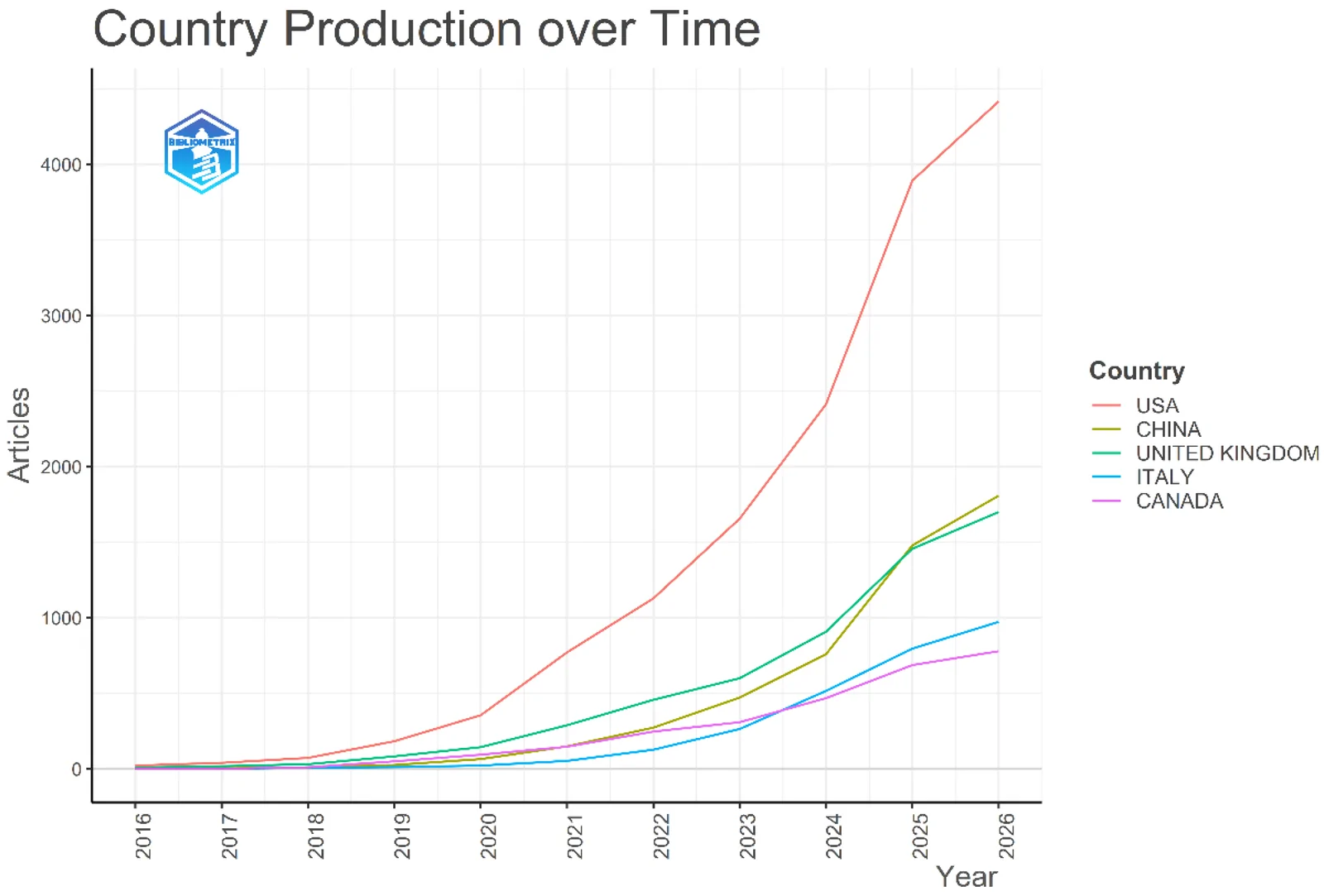
Country Production Over Time.

Scientific production by country (2016–2026) demonstrates the global expansion of AI governance and implementation research. The United States emerged as a major contributor, particularly after 2021, supported by substantial AI investments, a large healthcare market, and strong regulatory activity, including the highest number of FDA-approved AI/ML-based medical devices [39]. China ranked as the second-largest contributor after 2022, reflecting rapid growth in AI research capacity supported by national strategic investments, while the United Kingdom showed steady development. Italy and Canada also demonstrated increasing contributions, although at a smaller scale, with Italy showing accelerated growth after 2022. The country-level distribution presented in Figure 5 complements the overall publication trends in Figure 3 by highlighting the geographic factors driving research growth and confirming consistency between overall and national publication outputs.

#### 4.3.2. Institutional Contributions

**Figure 6:**
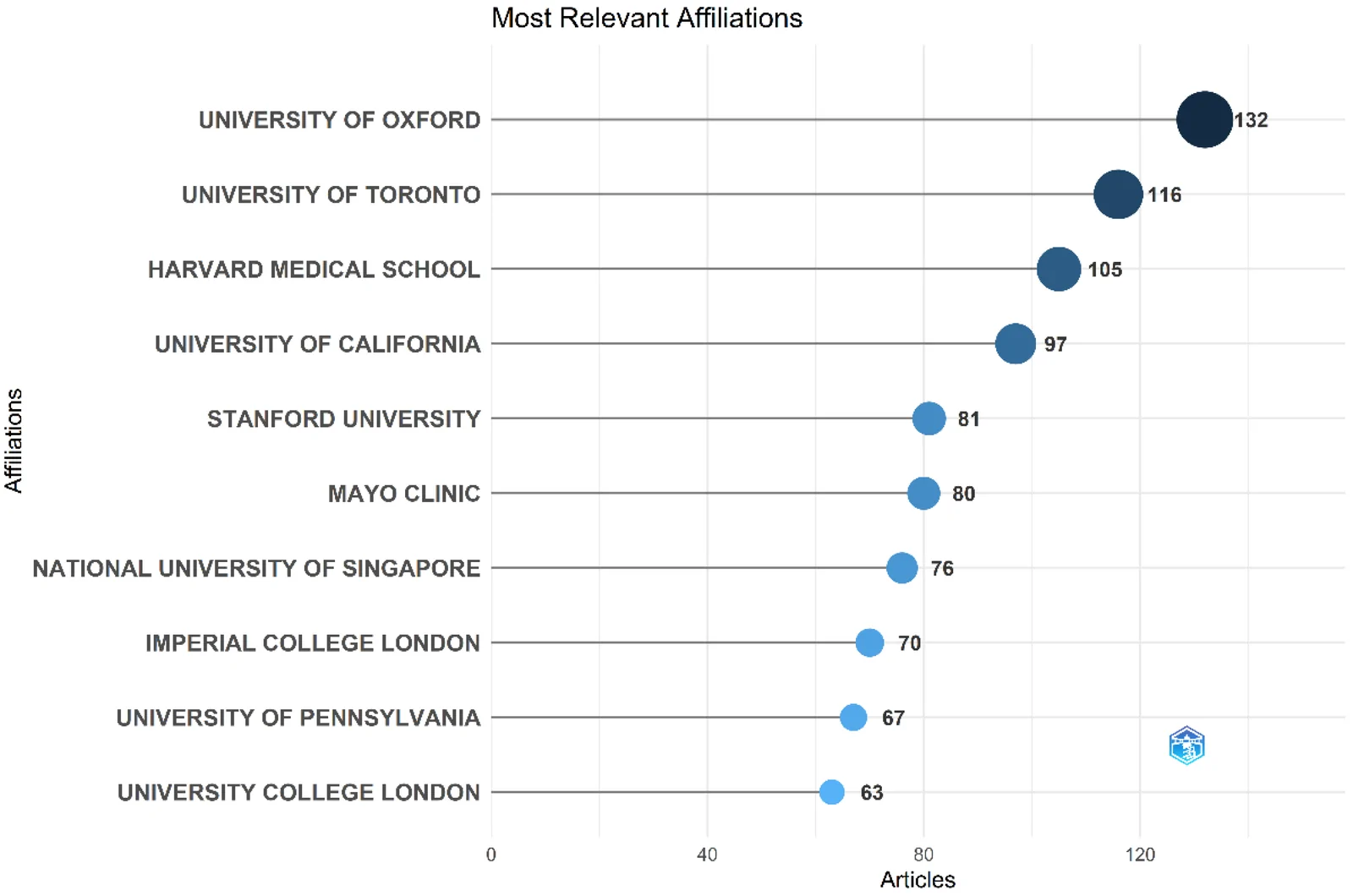
Most Relevant Affiliation.

The most productive institutional affiliations are presented in Figure 6. The University of Oxford is the top institution, followed by the University of Toronto, Harvard Medical School, University of California, Stanford University, Mayo Clinic, and Imperial College London. The dominance of these global institutions reflects their role in propelling the field forward, with most affiliations from the USA and UK due to robust research infrastructure and adequate financial resources.

#### 4.3.3. Individual Contributors

**Figure 7:**
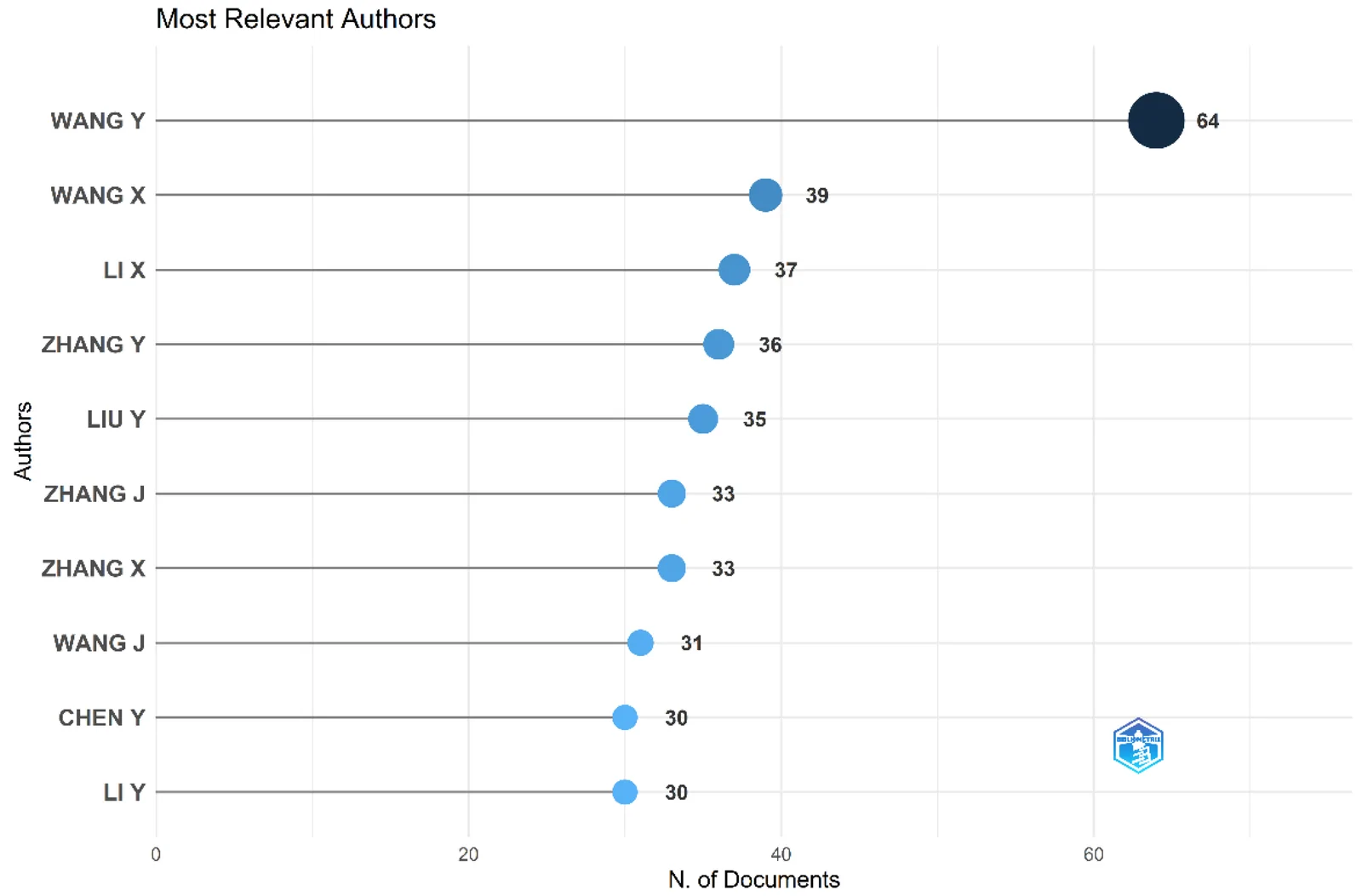
Most Relevant Authors.

The top authors by number of publications are shown in Figure 7. The small differences in publication counts indicate that research productivity is distributed among several authors rather than concentrated among a limited group. This pattern reflects the collaborative and interdisciplinary nature of the field.

#### 4.3.4. Journals and International Collaboration

The publications span 1500 sources across health policy, medical informatics, computer science, and ethics, reflecting the interdisciplinary nature of AI governance research. Although international collaboration is relatively high (34.66%), partnerships are mainly concentrated among high-income countries, with limited participation from developing countries. Nyamawe and Shao [14] noted that evidence regarding the effectiveness of governance mechanisms in clinical practice remains limited across different contexts. Similarly, Wei et al. [23] highlighted that regulatory frameworks differ across jurisdictions and that their practical implementation continues to face challenges.

#### 4.3.5. Most Relevant Source

As shown in Table 7, the top 10 sources account for 521 of the 3780 articles (13.78%). BMJ Open was the leading source, contributing 127 articles (3.36%). The remaining journals each contributed between 1.0% and 1.4%, indicating a relatively distributed publication pattern. The low concentration among the top sources suggests that research in this area is published across a wide range of journals.

#### 4.3.6. Countries Collaboration Map

As presented in Table 8, the US–UK pair ranked first with 170 collaborations (1.51%), reflecting strong research links between the two countries. The United States appeared in seven of the top 10 collaboration pairs, indicating its central role in international research networks. The United Kingdom also showed frequent collaborations with Italy, Germany, Australia, and India. The presence of China and India indicates increasing participation from Asian research communities. However, the relatively low percentages show that international collaboration is distributed across multiple country pairs, with no single partnership dominating the network.

### 4.4. Thematic Evolution

#### 4.4.1. Trend Topics over Time

**Figure 8:**
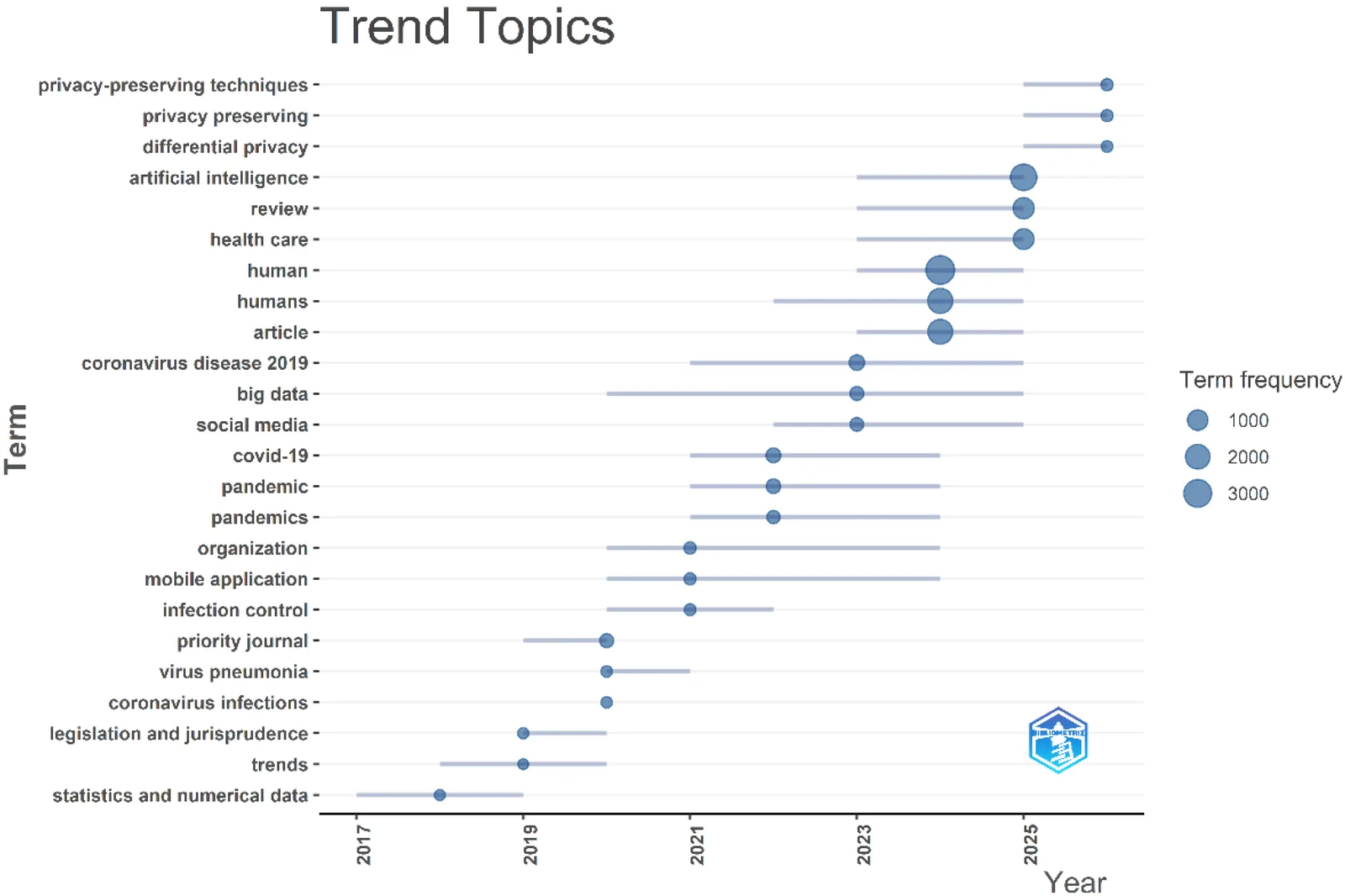
Trend Topic. Note: The figure was generated using Biblioshiny (R package). Each circle represents the median publication year of a term, and smaller circles indicate terms with lower publication frequency, while larger circles indicate higher frequency.

Figure 8 illustrates trends in key research topics between 2017 and 2026. As shown in Table 9, the most frequent terms are human (3331; 24.50%), artificial intelligence (2546; 18.73%), and humans (2153; 15.84%), indicating that the literature focuses on AI technologies and their human-related aspects. The terms article (2071; 15.23%) and review (1168; 8.59%) reflect the volume of published research, while health care (1066; 7.84%) highlights the field’s clinical focus. COVID-19 (188; 1.38%) and pandemic (157; 1.15%) had earlier median publication years (2022), indicating greater attention during the pandemic period. Big data (123; 0.90%) and social media (105; 0.77%) appeared less frequently, suggesting more specialized research areas. Overall, the findings indicate continued attention to AI in healthcare, with increasing interest in human-related issues and reduced emphasis on COVID-19-related research.

#### 4.4.2. Three Distinct Periods

Three distinct periods emerged from the temporal analysis, as presented in Table 10.

- Period 1 (2016–2019): The initial period was characterized by broad topics such as “statistics and numerical data,” “trends,” and “legislation and jurisprudence.” Research focused mainly on general ethical considerations without specific regulatory frameworks.
- Period 2 (2020–2022): The COVID-19 pandemic shifted attention toward direct clinical applications. Research priorities changed, with terms such as “coronavirus infection,” “viral pneumonia,” “pandemic,” and “infection control” becoming prominent. This period highlighted both the potential and risks associated with the rapid adoption of AI without corresponding governance frameworks [14].
- Period 3 (2023–2026): The most recent period reflects increased maturity, with growing attention to topics such as “privacy-preserving techniques,” “big data,” “differential privacy,” and specific governance mechanisms.

#### 4.4.3. Shift to Specific Regulatory Frameworks

Although research published between 2016 and 2019 rarely addressed specific regulations, publications from 2023 to 2026 increasingly referenced the EU AI Act (proposed in 2021 and enacted in 2024) and the FDA AI/ML Action Plan (released in 2021). This shift reflects a transition from general ethical discussions toward research focused on regulatory compliance.

- Evolution of Explainability: The treatment of explainability has evolved. Early research mainly considered explainability as an abstract principle. Recent studies, such as Amann et al. [43], examine explainability from multiple perspectives, including technological, legal, medical, and patient viewpoints, by exploring how it can be achieved and its potential benefits.
- Accountability: Increasing attention to terms such as “accountability,” “liability,” and “compliance” reflects growing recognition that ethical AI requires enforceable standards. This is consistent with Chau et al. [38], who identified unclear accountability and responsibility structures as major obstacles to implementation.

The thematic focus evolved from general ethical discussions (2016–2019) to pandemic-related applications (2020–2022), followed by increased attention to specific governance frameworks (2023–2026), particularly the FDA AI/ML pathway and the EU AI Act.

### 4.5. Proposed Integrated Governance Framework

**Figure 9:**
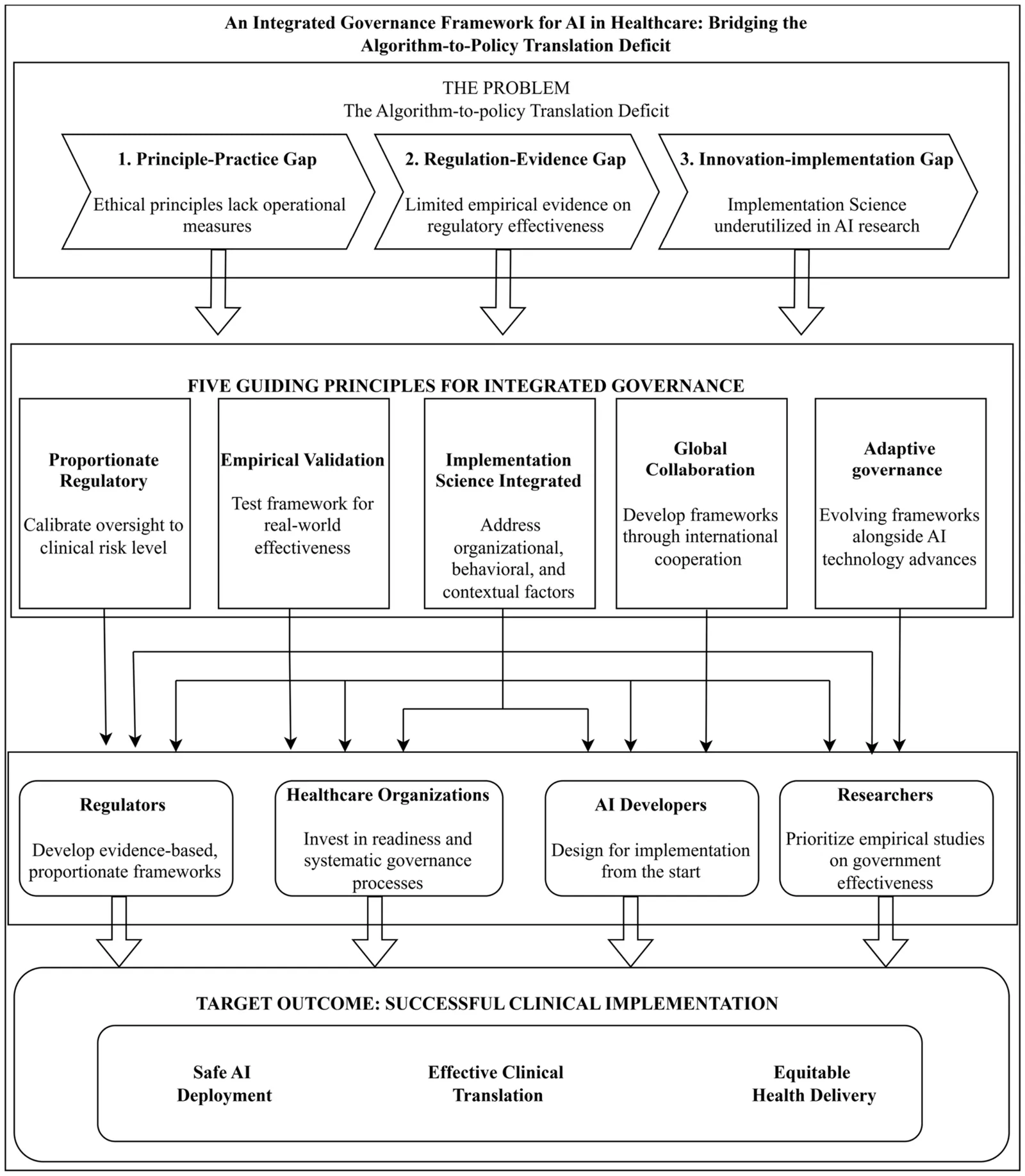
An Integrated Governance Framework for AI in Healthcare.

Based on the thematic analysis and identified research gaps, this study proposes an integrated governance framework to address the gap between algorithm development and policy implementation. Figure 9 presents the framework, which is structured around five guiding principles:

- Proportionate Regulation: Calibrating oversight to clinical risk level to avoid both under-regulation and over-regulation;
- Empirical Validation: Subjecting governance frameworks to rigorous real-world evaluation;
- Implementation Science Integration: Addressing organizational, behavioral, and contextual factors through established frameworks (CFIR, RE-AIM, PARIHS);
- Global Collaboration: Developing frameworks through international cooperation to ensure interoperability and diverse perspectives;
- Adaptive Governance: Designing frameworks that evolve alongside AI technological advances.

These principles are translated into specific roles for four stakeholder groups: regulators, healthcare organizations, AI developers, and researchers. The framework aims to support successful clinical implementation through safe AI deployment, effective clinical use, and equitable healthcare delivery. A detailed discussion of the framework is provided in Section 5.

## 5. Discussion

The discussion is organized into six thematic sections, each aligned with one or more of the five research questions guiding this study. Section 5.1 addresses RQ1 (publication growth trajectories), Section 5.2 addresses RQ2 (thematic clusters), Section 5.3 discusses RQ3 (key contributors and collaboration patterns), Section 5.4 examines RQ4 (thematic evolution), and Section 5.5 addresses RQ5 (research gaps). Section 5.6 synthesizes the findings and proposes an integrated governance framework that combines insights from the research questions. This structure ensures that each research question is addressed clearly and allows readers to identify the corresponding discussion section.

### 5.1. Publication Growth Trajectory and Governance Awareness

The annual growth rate of 47.65% in publications addressing AI governance and implementation reflects major changes in the research field. The initial period (2016–2019) showed limited publication activity, consistent with the “algorithm development phase,” where research focused mainly on technical validation [13]. The acceleration observed from 2020 onward corresponds with several developments, including the COVID-19 pandemic, which exposed limitations in existing governance structures, and the emergence of regulatory initiatives such as the EU AI Act and FDA guidance on AI/ML-based medical devices, which increased research attention toward governance and implementation.

This development is consistent with Benjamens et al. [39], who noted that “regulatory standards for AI-based medical devices vary widely across jurisdictions, indicating a lack of global harmonization.” The growth in publications suggests increasing research attention toward addressing regulatory fragmentation. The emergence of generative AI and large language models has further accelerated this trend, as existing governance frameworks were not designed for foundation models [46].

Although governance research grew by 47.65% annually, technical AI healthcare publications increased by approximately 35% during the same period [13]. However, governance-related publications (3780 documents) remain substantially fewer than technical AI research publications (estimated at more than 27,000 documents). These findings indicate that implementation science and governance research continue to lag behind technical development despite their rapid growth.

### 5.2. Thematic Structure: Technology Dominance and Governance Fragmentation

Thematic mapping shows that technical topics, particularly AI and machine learning, remain dominant, while governance-related topics such as ethics, privacy, and risk assessment are receiving increasing attention. Four main thematic groups were identified: regulatory compliance, which is the most developed area; ethical frameworks, which are conceptually developed but lack operational measures; organizational readiness, which remains underrepresented; and clinical workflow integration, which is the least developed area.

This dual structure is consistent with the findings of Morley et al. [35], who noted that ethical issues can be classified as (a) epistemic, related to misguided, inconclusive, or incomprehensible evidence; (b) normative, related to unfair outcomes and transformative effectiveness; or (c) related to traceability. However, the continued dominance of technical terms indicates that the field remains primarily focused on algorithm development rather than implementation. This aligns with the finding that “despite major technical advances, implementation of these models into clinical practice remains limited based on the available evidence” [13]. Governance and implementation themes are expanding but remain less prominent compared with technical AI research.

The presence of ethics and privacy as key themes indicates increasing attention to the normative aspects of AI healthcare research. However, Economou-Zavlanos et al. [9] noted that ethical principles are often discussed conceptually without clear methods for implementation in clinical systems. This gap remains a limitation, as research has identified ethical values but has not developed validated approaches for measuring compliance or supporting enforcement. The separation between technical, ethical, regulatory, and implementation themes reflects the fragmented nature of the field.

The dominance of human-centered terms, including human (24.50%), artificial intelligence (18.73%), and humans (15.84%), reflects the dual focus of the field on technological development and its implications for people. However, the limited frequency of terms such as privacy-preserving techniques and differential privacy, which appear with a median year of 2026, suggests that some governance mechanisms are only recently receiving attention. This finding is consistent with the identification of the principles–practice gap as a major limitation in the field.

### 5.3. Geographic and Institutional Concentration

The dominance of the United States, China, and the United Kingdom in publication output, along with the concentration of research activity among leading institutions, raises important questions about the generalizability of AI governance frameworks. The leading position of the United States reflects its large healthcare market, substantial AI research investments, and strong regulatory activity. Benjamin et al. [39] reported that the FDA has approved 64 AI/ML-based medical devices, representing the highest number among regulatory agencies.

China’s rapid growth after 2022 reflects national investments in AI research and development. However, Wei et al. [23] noted that “regulatory frameworks remain uneven across jurisdictions, and their implementation in practice continues to pose big challenges.” The concentration of research activity among well-resourced institutions reflects the resource-intensive nature of AI governance research and raises concerns about the diversity of perspectives represented in governance discussions. As Nyamawe and Shao [14] observed, evidence regarding the effectiveness of governance mechanisms in clinical practice remains limited across many contexts.

Although international collaboration is relatively high (34.66%), partnerships are mainly concentrated among high-income countries, with limited participation from developing countries. This pattern may lead to governance frameworks that reflect the priorities of wealthier nations while giving limited attention to the perspectives and needs of other regions. At the individual level, Wang Y, Wang X, and Zhang Y Anthony emerged as among the most productive authors. The publications span 1500 sources across health policy, medical informatics, computer science, and ethics, reflecting the interdisciplinary nature of the field. However, the concentration of research activities in a limited number of countries and institutions raises concerns about the generalizability of existing governance models.

Journal distribution further supports this pattern, with the Journal of Medical Internet Research (98 articles, 2.59%) and BMJ Open (127 articles, 3.36%) among the leading publication outlets. The presence of digital health and general medicine journals highlights the interdisciplinary scope of the field. However, the relatively low contribution of the top 10 sources, which collectively account for only 13.78% of the literature, indicates that AI governance research is distributed across many publication venues, reflecting its emerging and fragmented nature.

Country collaboration analysis shows that the US–UK pair ranks first (170 collaborations, 1.51%), with the United States appearing in seven of the top 10 collaboration pairs. This confirms the central role of Anglo-American institutions in shaping governance discussions. However, consistent with concerns raised by Nyamawe and Shao [14], such concentration may result in governance frameworks that reflect the priorities of high-income countries while overlooking the specific implementation challenges faced by developing countries.

In response to RQ3, the study summarizes the key contributors and collaboration patterns of AI healthcare governance research, as presented in Table 11.

The key finding for RQ3, summarized in Table 11, is that AI governance research is dominated by geographically concentrated networks of high-income institutions, which may limit the global applicability of existing frameworks. These implications are further examined through the principles-practice gap (Section 5.5) and addressed through the principle of global collaboration in the proposed governance framework (Section 5.6).

### 5.4. Thematic Evolution: From General Principles to Specific Frameworks

Temporal analysis shows a shift from broad discussions toward more focused governance issues. The initial period (2016–2019) mainly addressed general topics such as “statistics” and “legislation,” reflecting early efforts to examine the implications of AI. During the pandemic period (2020–2022), research attention shifted toward clinical applications, including disease monitoring and diagnosis. Recent studies (2023–2026) increasingly focus on topics such as “privacy-preserving techniques,” “big data,” and governance mechanisms. While research published between 2016 and 2019 rarely addressed specific regulations, publications from 2023 to 2026 increasingly reference the EU AI Act (proposed in 2021 and enacted in 2024) and the FDA AI/ML Action Plan (released in 2021).

This shift aligns with the increasing emphasis on operationalizing ethical principles. Oniani et al. [11] noted that “ethical principles of generative AI in healthcare remain understudied” and that “there are no clear solutions to address ethical issues.” The growing focus on privacy-preserving technologies and regulatory compliance reflects efforts to move from abstract ethical principles toward practical governance mechanisms.

Increasing attention to “accountability,” “liability,” and “compliance” reflects recognition that ethical AI requires enforceable standards. This is consistent with Chau et al. [38], who identified “unclear structures of accountability and responsibility” as “a major obstacle to implementation.” The increasing focus on specific governance issues indicates that researchers are addressing practical challenges associated with AI implementation.

The evolution of explainability is also evident. Early research mainly considered explainability as an abstract principle, whereas recent studies, such as Amann et al. [43], examine explainability through multiple perspectives, “considering how it can be achieved and what the benefits are from a development perspective.”

### 5.5. Critical Research Gaps: The Algorithm-to-Policy Translation Deficit

Three critical gaps contribute to the algorithm-to-policy translation deficit. Among these, the principles-practice gap appears the most urgent, as it challenges the practical value of governance discussions and leaves healthcare organizations with limited guidance for implementing ethical principles.

The identification of these gaps represents an interpretive synthesis of the bibliometric findings rather than direct measurements. The synthesis is based on three observations: (1) the limited representation of organizational readiness and clinical workflow integration themes; (2) the dominance of conceptual rather than empirical studies examining governance; and (3) the limited presence of implementation science frameworks within the keyword network. Therefore, these gaps are inferred from the relative prominence of research themes and should be further examined through empirical studies.

- Innovation–Implementation Gap: Implementation science remains underrepresented in AI healthcare research. Although established implementation frameworks, such as CFIR and RE-AIM, have been widely validated in healthcare innovation studies, their application to AI-focused research remains limited. Abogunrin et al. [34] highlighted that “regulatory considerations and health technology assessments are becoming critical components in determining whether AI systems can be implemented safely and effectively,” yet the integration of implementation science with AI governance remains at an early stage. This gap is particularly evident in generative AI. Meskó and Topol [46] noted that “existing governance frameworks weren’t designed for a platform model, resulting in a lag in regulation.” Current research on generative AI governance remains largely conceptual rather than empirical, leaving emerging issues such as hallucinations, explainability limitations, and unclear responsibilities insufficiently addressed.
- Principles–Practice Gap: The literature has developed extensive classifications of ethical values; however, validated approaches for measuring compliance and supporting enforcement remain limited. Pilkington et al. [12] noted that AI “impacts the doctor-patient relationship in a way that AI in other areas can’t,” yet few practical frameworks exist to maintain trust in clinical applications. Similarly, Vandersluis and Savulescu [24] argued that “the inclusion of underrepresented groups in algorithmic processes can result in harm,” while operational approaches for addressing algorithmic bias remain insufficiently defined and evaluated. The absence of operational guidance creates challenges for healthcare organizations seeking to implement AI responsibly. Economou-Zavlanos et al. [9] emphasized the need for “concrete guidelines for evaluating algorithmic technologies” that translate “key principles of ethics and quality” into measurable evaluation criteria. However, such guidelines remain limited and are not yet widely adopted in practice.
- Regulation–Evidence Gap: Empirical evidence regarding the effectiveness of regulatory approaches remains limited. Questions regarding how hospitals implement EU AI Act requirements or the challenges faced by clinicians using FDA-approved AI tools remain largely unanswered. This gap is concerning because regulatory frameworks are being developed without sufficient evidence synthesis and evaluation. The findings support Abogunrin et al. [34], who noted that “the acceptability of AI-assisted evidence synthesis by HTA bodies remains underexplored.” Similarly, Graham and Nur Akcan [45] found that “the announcement of the EU AI Law didn’t lead to a big change in the number of devices approved in the EU, contrary to previous research.” These findings highlight the need for evidence-based regulatory development, where governance frameworks are systematically evaluated to ensure effectiveness and avoid unintended consequences.

Table 12 summarizes the evidence from the bibliometric analysis and the implications of these three critical gaps for AI governance and implementation research.

### 5.6. Toward Integrated Governance Framework

Figure 9 presents the integrated governance framework proposed in this research. This framework is a conceptual model derived from bibliometric evidence and literature synthesis, designed to organize key governance principles and guide future research and practice. It is not intended as a validated clinical intervention; rather, the proposed principles require further evaluation through prospective implementation studies to assess their feasibility and effectiveness in real-world settings.

This framework addresses the algorithm-to-policy translation deficit, which consists of three interconnected gaps: the principles-practice gap, where ethical principles lack operational measures; the regulations-evidence gap, where empirical evidence on the effectiveness of regulatory approaches remains limited; and the innovation-implementation gap, where implementation science is underutilized in AI research.

To bridge this gap, the framework is structured around five evidence-based principles:

- Proportional Regulation: Governance approaches should be tailored to the level of clinical risk, avoiding excessive regulation that may limit innovation and insufficient regulation that may allow unsafe implementation. Bignami et al. [44] emphasized that the “risk-based classification” approach in the EU AI Act recognizes that “risk thresholds are arbitrary, not based on evidence,” highlighting the need for flexible regulatory approaches.
- Empirical Validation: Governance frameworks should undergo rigorous empirical evaluation. Graham and Nur Akcan [45] found that the introduction of the EU AI Act did not result in statistically significant changes in approved devices, demonstrating the importance of evaluating whether regulatory approaches achieve their intended outcomes.
- Integration of Implementation Science: Governance frameworks should incorporate organizational, behavioral, and contextual factors that influence AI adoption. Lee and Yoon [67] found that “AI tools in clinical settings often fail beyond the pilot stage due to weak organizational readiness.” Therefore, implementation science frameworks such as CFIR, RE-AIM, and PARIHS should be adapted to address the specific challenges of AI implementation.
- Global Collaboration: Governance frameworks should be developed through international collaboration that incorporates diverse perspectives and implementation contexts. Wei et al. [23] identified a “global shift towards a hybrid and increasingly interoperable regulatory model,” highlighting the importance of international cooperation in developing effective governance approaches.
- Adaptive Governance: Governance frameworks should evolve alongside advances in AI technologies. Meskó and Topol [46] argued that “existing governance frameworks weren’t designed for the foundation model,” emphasizing the need for adaptive mechanisms, including regulatory sandboxes and real-world evidence generation, to maintain effectiveness as AI technologies develop.

These five principles are derived directly from the bibliometric findings: fragmented regulatory standards support the need for proportional regulation; the dominance of conceptual studies over empirical research highlights the importance of empirical validation; limited attention to organizational readiness and clinical workflow integration emphasizes the need for implementation science integration; the concentration of research activity in high-income countries demonstrates the importance of global collaboration; and the rapid evolution of AI technologies and governance themes supports the need for adaptive governance. Table 13 provides a direct mapping between the bibliometric findings and the proposed governance principles.

These principles are operationalized through the roles and responsibilities of four main stakeholder groups:

- Regulators should develop proportional and evidence-based governance frameworks through systematic consultation with implementation science experts and establish mechanisms for evaluating regulatory effectiveness.
- Healthcare organizations should invest in organizational readiness, including staff training, infrastructure development, and change management. They should also establish systematic governance processes for AI evaluation, procurement, and implementation through multidisciplinary committees.
- AI developers should consider implementation requirements from the early stages of development by prioritizing transparency and explainability and generating evidence on implementation outcomes in addition to technical performance.
- Researchers should integrate implementation science frameworks into AI studies, prioritize empirical evaluations of governance effectiveness, and promote interdisciplinary collaboration across technical, clinical, ethical, and implementation domains.

This framework culminates in the targeted outcome of successful clinical implementation, characterized by safe AI deployment, effective clinical translation, and equitable healthcare delivery. However, these outcomes represent theoretical objectives that require empirical validation. The framework provides a conceptual structure for organizing governance principles, but its effectiveness must be evaluated through prospective implementation studies and real-world assessments.

The continuous feedback loop illustrated in Figure 9 represents the application of adaptive governance (Principle 5), emphasizing continuous learning and improvement based on real-world evidence. This mechanism aims to ensure that the governance structure remains responsive and effective as AI technologies continue to evolve.

## 6. Conclusions

This bibliometric analysis examines the intellectual development of AI healthcare research at the intersection of implementation science and governance frameworks during the 2016–2026 period, showing that the field is undergoing rapid but structurally fragmented growth.

The analysis identifies a 47.65% annual growth rate in governance-focused publications, exceeding the growth rate of technical AI research. However, governance-related publications (3780 documents) remain substantially fewer than technical AI publications, confirming that implementation science and governance research continue to lag behind technical development. Four dominant thematic groups were identified: regulatory compliance as the most developed theme; ethical frameworks that are conceptually advanced but lack operational measures; organizational readiness, which remains underrepresented; and clinical workflow integration, which is the least developed area. These findings confirm the structural fragmentation identified in the literature.

Geographically, the United States leads research output, followed by China and the United Kingdom. Leading institutions include Oxford University, the University of Toronto, Harvard Medical School, Stanford University, and the Mayo Clinic, while Wang Y, Wang X, and Zhang Y were among the most productive individual contributors. Despite a relatively high level of international collaboration (34.66%), limited participation from developing countries raises concerns about the global applicability and generalizability of existing AI governance frameworks.

The journal distribution shows that the Journal of Medical Internet Research (98 articles, 2.59%) and BMJ Open (127 articles, 3.36%) are among the leading publication outlets. However, the top 10 journals collectively account for only 13.78% of the literature, reflecting the dispersed and interdisciplinary nature of AI governance research. Thematic evolution progressed through three periods: general ethical and conceptual topics, pandemic-driven clinical applications, and increased attention to specific regulatory frameworks, with growing references to the EU AI Act and the FDA AI/ML Action Plan.

Analysis of trending topics shows that the most frequent terms are human (24.50%), artificial intelligence (18.73%), and humans (15.84%). This pattern indicates that the literature focuses on both AI technologies and their implications for human-centered healthcare.

Three critical gaps contribute to the algorithm-to-policy translation deficit. The principles–practice gap represents the most immediate challenge, as ethical taxonomies have advanced but lack validated methods for measurement and implementation. The regulations–evidence gap remains a major concern due to limited empirical evidence regarding the effectiveness of regulatory approaches. The innovation–implementation gap represents a longer-term challenge, as implementation science remains underrepresented in AI healthcare research. The emergence of generative AI introduces additional challenges that influence all three gaps.

To address this fragmentation, this study proposes an integrated governance framework guided by five principles: proportional regulation, empirical validation, integration of implementation science, global collaboration, and adaptive governance. Derived from bibliometric evidence, this conceptual framework aims to bridge the gap between algorithm development and policy-guided clinical practice by guiding future research and empirical validation. However, its operational effectiveness requires systematic evaluation under real-world clinical conditions.

This study has several limitations, including reliance on a single database (Scopus), inclusion of English-language publications only, exclusion of policy documents and conference proceedings, and dependence on author keywords, which may not fully capture the conceptual scope of the literature. In addition, although the study period covered 2016–2026, the literature search was conducted in May 2026. Therefore, publications from 2026 may be incomplete and should be interpreted with caution.

This search strategy did not explicitly include specific cybersecurity, blockchain-enabled healthcare optimization, secure data sharing technologies, or trust-based computing categories, which may have resulted in underrepresentation of security-focused AI governance literature. These areas were beyond the scope of this bibliometric analysis. Future research should incorporate these dimensions to provide broader coverage of cybersecurity, privacy-preserving technologies, and secure AI governance frameworks.

Future studies could also address methodological limitations by combining multiple databases, such as PubMed and other indexing platforms, and incorporating additional sources of evidence to improve the completeness and validation of findings. Addressing these limitations will support a more integrated and evidence-based understanding of AI governance and its role in improving healthcare outcomes while reducing risks and promoting equity.

## Figures and Tables

**Table 1:** Evolution of AI Healthcare Research (2016–2026).

Period	Number of Studies	Dominant Focus	Key Characteristics	Limitation
2016–2019	148	Algorithm Development	Model accuracy, diagnostics, imaging	No governance or implementation linkage
2020–2022	730	Ethical and Governance Awareness	Fairness, privacy, explainability	Remains conceptual, lacks operational tools
2023–2026	2902	Implementation and Policy Integration	Regulation, clinical adoption, real-world use	Limited empirical validation; fragmented across domains

**Table 2:** Key Governance Principles in AI Healthcare.

Principle	Description	Evidence from Literature	Operational Gap
Transparency	Explainability of AI decisions	Amann et al. [43]	No validated measure of sufficient transparency
Accountability	Clear responsibility for outcomes	Chau et al. [38]	Unclear how to assign liability in practice
Human Oversight	Clinician involvement in decisions	Abogunrin et al. [34]	No standard for meaningful vs. token oversight
Risk-Based Regulation	Classification based on risk level	Bignami et al. [44]; Graham and Nur Akcan [45]	Risk thresholds are arbitrary, not evidence-based
Fairness and Bias Mitigation	Preventing algorithmic discrimination	Vyas et al. [36]; Morley et al. [35]	Multiple fairness definitions, no consensus

**Table 3:** Structural Fragmentation in AI Healthcare Research.

Domain	Main Focus	Maturity Level	Key Limitation
Technical AI Research	ML models, prediction systems	Highly developed	Weak or absent link to clinical governance
Ethical AI Research	Fairness, transparency, bias	Highly developed	Lack of operationalization and measures
Governance and Regulation	FDA, EU AI Act, compliance systems	Emerging	Limited empirical validation in practice
Implementation Science	Adoption frameworks (CFIR, RE-AIM)	Underdeveloped in AI context	Poor integration with AI-specific studies
Organizational Readiness	Workflow integration, institutional capacity	Underexplored	Limited real-world evidence
Generative AI Governance	LLM safety, hallucination risks	Very recent	No standardized or validated frameworks

**Table 4:** Barriers to AI Implementation in Healthcare.

Category	Specific Barrier	Supporting Evidence	Nature of Barrier
Technical	Lack of interoperability with existing systems	Zhou et al. [31]	Structural
Organizational	Limited expertise and inadequate infrastructure	Abogunrin et al. [34]	Capacity-based
Legal	Unclear liability and accountability frameworks	Chau et al. [38]	Regulatory
Clinical	Workflow misalignment and clinician resistance	Vallée [30]	Behavioral
Regulatory	Fragmented standards across jurisdictions	Benjamens et al. [39]	Policy-based
Technical/Security	Limited integration of privacy-preserving technologies (blockchain, federated learning) with governance frameworks	Alaskar et al. [24]; Ben Gara Ali and Smiti [5]	Implementation-based

**Table 5:** Comparative Analysis of Key Studies in AI Healthcare Governance Research.

Study	Focus	Methodology	Domain	Framework Addressed	Limitation
Aggarwal et al. [13]	Technical AI in medical imaging	Systematic review and meta-analysis	Technical AI	None	No governance linkage
Morley et al. [35]	Ethical AI in healthcare	Mapping review	Ethical AI	Fairness, transparency, accountability	Lack operational measures
Vyas et al. [36]	Algorithmic bias	Conceptual analysis	Ethical AI	Fairness, bias mitigation	No implementation pathway
Benjamens et al. [39]	FDA-approved AI devices	Database analysis	Regulatory	FDA approval pathways	Descriptive only
Amann et al. [43]	Explainability in healthcare	Multidisciplinary review	Governance	Transparency, explainability	No validation framework
Abogunrin et al. [34]	AI adoption in health technology assessment	Systematic review	Implementation	HTA, regulatory compliance	No implementation framework
Graham and Nur Akcan [45]	EU AI Act impact	Quantitative analysis	Regulatory	EU AI Act	Limited to EU context
Meskó and Topol [46]	Generative AI governance	Conceptual analysis	Governance	Generative AI oversight	No operational guidance
Economou-Zavlanos et al. [9]	Ethical and quality principles	Framework development	Ethical AI	Ethical evaluation criteria	No empirical validation
Chau et al. [38]	AI liability and accountability	Conceptual analysis	Legal	Accountability frameworks	No implementation evidence
Present Study	AI governance and implementation	Bibliometric analysis	Integrated governance	Comprehensive governance framework	Reliance on single database

Note: Studies categorized as ‘Governance’ or ‘Regulatory’ domains primarily propose conceptual frameworks. Studies evaluating the empirical effectiveness of these frameworks in clinical settings remain absent from the literature.

**Table 6:** Dominant Thematic Clusters in AI Governance Research.

Cluster	Development Level	Key Characteristics
Regulatory Compliance	Most developed	FDA approval pathways, EU AI Act, HTA
Ethical Frameworks	Conceptually developed	Fairness, transparency, accountability
Organizational Readiness	Underrepresented	Infrastructure, training, change management
Clinical Workflow Integration	Least developed	Implementation science, adoption

**Table 7:** Most Influential Journals in AI Governance Research (2016–2026).

Source	Articles	Percentage (%)
BMJ OPEN	127	3.36
JOURNAL OF MEDICAL INTERNET RESEARCH	98	2.59
FRONTIERS IN DIGITAL HEALTH	53	1.40
INTERNATIONAL JOURNAL OF ENVIRONMENTAL RESEARCH AND PUBLIC HEALTH	51	1.35
CANCERS	41	1.08
NPJ DIGITAL MEDICINE	41	1.08
INTERNATIONAL JOURNAL OF MEDICAL INFORMATICS	40	1.06
JOURNAL OF CLINICAL MEDICINE	38	1.01
FRONTIERS IN MEDICINE	32	0.85

**Table 8:** Top 10 Country Collaboration Pairs.

Country Pair	Frequency	Percentage (%)
USA–UNITED KINGDOM	170	1.51
USA–CANADA	104	0.93
USA–GERMANY	97	0.86
USA–ITALY	78	0.69
UNITED KINGDOM–ITALY	74	0.66
USA–CHINA	70	0.62
UNITED KINGDOM–GERMANY	67	0.60
USA–AUSTRALIA	63	0.56
UNITED KINGDOM–AUSTRALIA	61	0.54
USA–INDIA	61	0.54

**Table 9:** Top 10 Trending Topics.

Term	Frequency	Percentage (%)	Year (Median)
human	3331	24.50	2024
artificial intelligence	2546	18.73	2025
humans	2153	15.84	2024
article	2071	15.23	2024
review	1168	8.59	2025
health care	1066	7.84	2025
COVID-19	188	1.38	2022
pandemic	157	1.15	2022
big data	123	0.90	2023
social media	105	0.77	2023

**Table 10:** Thematic Evolution Periods in AI Governance Research (2016–2026).

Period	Dominant Themes	Characteristics
2016–2019	Statistics, legislation, jurisprudence	General topics, initial efforts
2020–2022	Coronavirus, pandemic, infection control	COVID-19 focus
2023–2026	Privacy-preserving, differential privacy, big data	Specific governance mechanisms

**Table 11:** Key Contributors and Collaboration Patterns.

Dimension	Key Findings	Implications
Leading Countries	USA, China, UK (top 3)	Governance priorities reflect high-income contexts
Top Institutions	Oxford, Toronto, Harvard, Stanford, Mayo	Elite academic center perspective dominates
Leading Authors	Wang Y, Wang X, Zhang Y, Anthony	Distributed productivity, interdisciplinary background
Core Journals	JMIR, BMJ Open, Frontiers in Digital Health	Interdisciplinary audience; informatics/clinical focus
Collaboration Intensity	34.66% international collaboration	Moderate international engagement
Collaboration Geography	USA-UK most frequent; high-income concentration	Limited developing country participation
Network Structure	USA central hub; European secondary nodes	Fragmented network with global north dominance

**Table 12:** Major Research Gaps in AI Governance and Implementation Research.

Gap	Evidence from Bibliometric Analysis	Implication
Innovation-Implementation Gap	Organizational readiness and workflow integration are underdeveloped clusters	Poor translation into practice
Principle-Practice Gap	Ethics themes remain conceptual	Lack of operational governance
Regulation-Evidence Gap	Few empirical studies evaluating regulations	Limited evidence for policy effectiveness

**Table 13:** Mapping Bibliometric Findings to Governance Framework Principles.

Bibliometric Finding	Derived Principle	Rationale
Regulatory compliance most developed, but fragmented across jurisdictions	Proportionate Regulation	Need for calibrated, harmonized oversight
Regulation-evidence gap: few empirical studies evaluating governance effectiveness	Empirical Validation	Governance frameworks require real-world testing
Organizational readiness and workflow integration are underdeveloped clusters	Implementation Science Integration	Adoption depends on behavioral and contextual factors
Geographic concentration in high-income countries; limited developing nation participation	Global Collaboration	Diverse perspectives essential for generalizability
Thematic evolution shows rapid shifts; generative AI creates novel governance challenges	Adaptive Governance	Frameworks must evolve with technological change

## Data Availability

The data used in this study were obtained from publicly available Scopus bibliometric records. No supplementary materials are associated with this manuscript.
